# Higher Mortality and Intensive Care Unit Admissions in COVID-19 Patients with Liver Enzyme Elevations

**DOI:** 10.3390/microorganisms8122010

**Published:** 2020-12-16

**Authors:** Lucia Taramasso, Antonio Vena, Francesca Bovis, Federica Portunato, Sara Mora, Chiara Dentone, Emanuele Delfino, Malgorzata Mikulska, Daniele Roberto Giacobbe, Andrea De Maria, Laura Magnasco, Mauro Giacomini, Antonio Di Biagio, Matteo Bassetti

**Affiliations:** 1 Infectious Disease Clinic, IRCCS Policlinic San Martino Hospital, 16132 Genoa, Italy; antonio.vena@hsanmartino.it (A.V.); fedu90@gmail.com (F.P.); chiara.dentone@hsanmartino.it (C.D.); Emanuele.delfino@hsanmartino.it (E.D.); daniele.roberto.giacobbe@gmail.com (D.R.G.); 2 Biostatistics Unit, Department of Health Sciences, University of Genoa, 16132 Genoa, Italy; francesca.bovis@gmail.com; 3 Department of Mental Health and Public Medicine, Section of Infectious Diseases, University of Campania, 80138 Naples, Italy; 4 Department of Informatics, Bioengineering, Robotics and System Engineering (DIBRIS), University of Genoa, 16145 Genoa, Italy; sara.mora.1994@gmail.com (S.M.); Mauro.Giacomini@dibris.unige.it (M.G.); 5 Infectious Disease Clinic, Department of Health Sciences (DISSAL), University of Genoa, 16132 Genoa, Italy; m.mikulska@unige.it (M.M.); 55225@unige.it (A.D.M.); lmagnasco90@gmail.com (L.M.); antonio.dibiagio@hsanmartino.it (A.D.B.); Matteo.Bassetti@hsanmartino.it (M.B.); 6 Centre of Excellence for Biomedical Research (CEBR), University of Genoa, 16132 Genoa, Italy

**Keywords:** liver enzyme elevations, COVID-19, SARS-CoV-2, mortality, intensive care unit

## Abstract

The aim of the present study is to evaluate if an independent association exists between liver enzyme elevations (LEE) and the risk of mortality or intensive care unit (ICU) admissions in patients with COVID-19. This was a single-center observational study, recruiting all consecutive adults with COVID-19. The elevation of aspartate aminotransferase (AST) or alanine aminotransferase (ALT) to the highest level between COVID-19 diagnosis and hospital discharge was categorized according to a standardized toxicity grade scale. In total, 799 patients were included in this study, 39% of which were female, with a mean age of 69.9 (±16.0) years. Of these patients, 225 (28.1%) developed LEE of grade ≥2 after a median of three days (interquartile range (IQR): 0–8 days) from the diagnosis of COVID-19, and they were estimated to have a higher hazard of death or ICU admission (adjusted hazard ratio (aHR): 1.46, 95% confidence interval (CI): 1.14–1.88). The clinical and laboratory variables associated with the development of LEE were male sex, higher respiratory rate, higher gamma glutamyl transpeptidase (GGT) and lower albumin levels at baseline. Among the analyzed treatments, steroids, tocilizumab and darunavir/ritonavir correlated with LEE. In conclusion, LEE were associated with mortality and ICU admission among COVID-19 patients. While the origin of LEE is probably multifactorial, LEE evaluation could add information to the clinical and laboratory variables that are commonly evaluated during the course of COVID-19.

## 1. Introduction

In last few months, Severe Acute Respiratory Syndrome Coronavirus 2 (SARS-CoV-2), the etiological agent of COVID-19, has caused an increasing number of respiratory illness worldwide [1,2]. During the pandemic, it has become evident that patients with COVID-19 did not only experience respiratory illness, but also immunologic dysregulation [3] and heart [4], kidney [5] and liver [6,7] involvement in a systemic disease involving multiple organs. In this context, it has been a common experience to deal with patients with abnormal liver enzyme levels, as it is estimated that about 46% of hospitalized COVID-19 patients have elevated plasma aspartate aminotransferase (AST) and 35% have elevated alanine aminotransferase (ALT) levels already at the time of hospital admission [8]. However, the pathogenesis of liver enzyme elevation (LEE) is not completely understood, although multiple causes have been hypothesized to explain the phenomenon, such as direct viral liver or muscle injury [6,9], viral binding to angiotensin-converting enzyme 2 (ACE2)-positive cholangiocytes [10], hepatic congestion secondary to high positive end expiratory pressure during mechanical ventilation [11], drug-induced toxicity [11], liver hypoxic damage in the course of multiorgan failure [12], hepatic damage from immune interactions involving intrahepatic cytotoxic T cells and Kupffer cells [11], and the co-existence of more of these conditions at the same time in the more severe cases of COVID-19.

Higher AST levels were found in patients with refractory COVID-19 pneumonia and liver damage indices, including AST and ALT, were higher in those with acute respiratory distress (ARDS) compared to others in China [13,14]. In a previous publication, we described the first 317 cases of COVID-19 followed in our center, and we found that 32% of patients had AST >40 U/L at first clinical presentation and that this finding was more frequent in patients who died compared to survivors [15]. Many other studies have now confirmed that AST or ALT elevation is more common in the more severe forms of COVID-19 [9,16] and it correlates with a poorer prognosis [17,18], even if some discordant results have also been reported [9,16,19,20]. However, data on factors associated with LEE, including possible pre-existing liver disease and exposure to the drugs used in the first phase of the pandemic of COVID-19, are still lacking [8,21,22,23,24]. Moreover, most data are extrapolated from Chinese [17,20] or American [25,26] studies, while little data have been published so far in the European population [18]. The aim of the present study was to evaluate if an independent association exists between LEE and the risk of mortality or ICU admission in a large cohort patients with COVID-19 followed up in Italy. The secondary aim was to identify the clinical, therapeutic and laboratory factors independently associated with LEE.

## 2. Materials and Methods

This was a single-center, retrospective, observational study, recruiting all consecutive adults with laboratory confirmed SARS-CoV-2 infection diagnosed from 25 February 2020 to 23 April 2020 at our 1200-bed tertiary hospital (Istituto di Ricovero e Cura a Carattere Scientifico-IRCCS Policlinico San Martino) in Genoa, Italy.

A confirmed case of COVID-19 was defined by a positive result on reverse transcriptase-polymerase chain reaction (RT-PCR) assay of a specimen collected on a respiratory sample (nasopharyngeal swabs, sputum or bronchoalveolar lavage). The date of the first positive SARS-CoV-2 sample was considered the baseline for this study. All laboratory-confirmed episodes of COVID-19 were recorded in a prospective registry carried out by a multidisciplinary group dedicated to the management of COVID-19 [15,27]. Patients for whom transaminase values were not available were excluded from the study. No other exclusion criteria were applied. The following data were collected from the patients’ medical records at the time of the first SARS-CoV-2 positive sample: age in years; gender; baseline underlying disease (both separately and summarized by means of the Charlson Comorbidity Index) and chronic therapies; date of illness onset; respiratory rate in breaths per minute; blood pressure; blood and serum laboratory results (white blood count, platelet count, ALT in U/L, AST in U/L, gamma glutamyl transpeptidase (GGT) in U/L, interleukin-6 (IL-6) in ng/mL, C-reactive protein (CRP) in mg/L, partial pressure of oxygen (PaO_2_)). During the course of hospitalization, data about treatments (antivirals, hydroxychloroquine, antibiotics, corticosteroids and tocilizumab), intensive care unit (ICU) admission with need for invasive mechanical ventilation, and the development of LEE were also collected. The elevation of AST or ALT to the highest level between COVID-19 diagnosis and hospital discharge was categorized according to a standardized toxicity grade scale [28,29,30]. Patients were classified based on changes relative to the upper limit of normal (ULN) of the reference laboratory, i.e., 40 U/L for both AST and ALT [31]: grade 0, less than 1.25 ULN; grade 1, 1.25–2.4× ULN; grade 2, 2.5–5× ULN; grade 3, 5.1–10× ULN; grade 4, >10× ULN (Table 1).

### 2.1. Statistical Methods

The primary endpoint of the analysis was a composite of mortality and ICU admission in patients with a LEE of grade ≥2. The secondary endpoint was grade ≥2 LEE. The baseline of the analysis was the first positive SARS-CoV-2 sample and each patient was followed until death or last available follow up. The follow up time was censored on 24 July 2020, six months after the study start date. The incidence rate of LEE was calculated as the number of new cases over person-months of follow up at risk. Crude hazard ratio (HR) of death or ICU admission was calculated for LEE grade 2, grade 3 and grade 4 through a Cox analysis. To estimate the causal hazard ratio of death or ICU admission according to grade ≥2 LEE, we fitted a weighted Cox regression model adjusting for variables that reached a *p* value <0.1 at univariate analysis, using a stepwise procedure. The same statistical method was then used for the secondary analysis conducted to estimate the hazard ratio of grade ≥2 LEE in the study population. Finally, a sensitivity analysis was also performed through a Cox analysis to investigate the HR for mortality in patients with LEE (Table S1).

To make efficient use of the available data, we used an advanced multiple imputation of missing values strategy using the proc MI multiple imputation procedure (10 imputations).

All the analyses were repeated in the subgroup of patients with complete available data to exclude potential confounding due to imputation and the main results were confirmed (data not shown).

All analyses were carried out using SAS software version 9.4 (Institute Inc., Cary, NC, USA).

### 2.2. Ethics

The collection of anonymized data for the present study was approved by the local ethics committee (Liguria Region Ethics Committee, registry number 163/2020), and specific informed consent was waived due to the retrospective nature of the study.

## 3. Results

### 3.1. Study Population

During the study period, 864 patients were diagnosed with SARS-CoV-2 infection. Of them, 65 had no blood tests available for LEE evaluation and were thus excluded. The 799 patients included in the study were 39.2% female (*n* = 313), with a mean age of 69.9 (±16.0) years. Four hundred and forty-two of them (66.7%) had at least one comorbidity. The more frequent comorbidities were hypertension (48.9%, *n* = 332), peripheral vasculopathy (19.6%, *n* = 131) diabetes mellitus (15.3%, *n* = 104), cerebrovascular disease (13.8%, *n* = 92), chronic obstructive pulmonary disease (COPD) (11.5%, *n* = 77), solid cancer (11.1%, *n* = 74), chronic kidney disease (10.9%, *n* = 72) and ischemic heart disease (9.5%, *n* = 64). Only one patient had known Hepatitis B surface antigen (HBsAg) positivity and 11 had positive Hepatitis C virus antibodies (HCV-Ab). At baseline, ALT and AST mean values were 43 (±69.1) and 49 (±64.0) U/L (Table S2). The baseline characteristics of the study population are further detailed in Table 2.

### 3.2. Mortality and ICU Admission in Patients with LEE

Two hundred and twenty-five patients (28.1%) developed LEE of grade ≥2 during the study period, with an estimated incidence rate of 10.5/100 patient month follow up (PMFU). LEE grade ≥3 and LEE grade 4 were seen in 76 and 22 patients, respectively, with an estimated incidence of 3.54 and 1.03/100 PMFU. The median time between the diagnosis of COVID-19 and the development of LEE (of any grade) was three days (interquartile range (IQR): 0–8 days). At univariate analysis (Table 3), LEE of grade ≥2 correlated with death or ICU admission with a HR of 1.45 (95% confidence interval (CI): 1.17–1.82, *p* = 0.0009). The HR increased to 1.82 (95% CI: 1.34–2.47) and 2.64 (95% CI: 1.66–4.19) when LEE of grade ≥3 or grade 4 were analyzed (*p* = 0.0001 and *p* < 0.0001, respectively). After adjusting for the main confounders, patients with LEE grade ≥2 still had a higher hazard of death or ICU admission when compared with patients without LEE (adjusted hazard ratio, aHR, 1.46, 95% CI: 1.14–1.88, Table 3). The additional clinical variables retained in the final multivariable model that showed a correlation with the composite outcome were age, (aHR 1.03 for each year increase, 95% CI: 1.02–1.04), male sex (aHR 1.32, 95% CI: 1.04–1.68) and higher Charlson comorbidity index (aHR 1.07 for each point increase, 95% CI: 1.04–1.12). Baseline laboratory parameters indicative of a higher state of inflammation were also associated with the outcome, namely higher baseline levels of white blood cells, CRP, IL-6, ferritin and longer prothrombin time (Table 3). Among the considered treatments, only steroids showed a protective hazard ratio (aHR 0.73, 95% CI: 0.58–0.91), while darunavir/ritonavir use had an aHR of 1.32 (95% CI: 1.01–1.71). No differences were found in patients treated with or without hydroxychloroquine, lopinavir/ritonavir, tocilizumab or antibiotics.

In a sensitivity analysis considering the HR for mortality, only LEE of grade 4 correlated with the outcome, while LEE of lower grades were not (see Table S1).

### 3.3. Factors Associated with LEE (Grade ≥2)

Several factors were associated with LEE of grade ≥2 in the univariate analysis (Table 4). The clinical variables that maintained a significant association after adjustment for confounders were male sex (aHR 1.73, 95% CI: 1.26–2.38), and respiratory rate (aHR 1.01 for each one-point increase, 95% CI: 1.00–1.02). At baseline, higher GGT and lower albumin levels were associated with a higher risk of LEE. Among the analyzed treatments, steroids, tocilizumab and darunavir/ritonavir correlated with LEE of grade ≥2 (Table 4).

A higher baseline Charlson Comorbidity Index did not correlate with a higher risk of LEE and was instead protective (Table 4). To further investigate this finding, we separately analyzed the role of the major comorbidities included in the evaluation of this score, and found that patients with diabetes mellitus with end-organ damage, chronic kidney disease or peripheral vascular disease had a lower risk of LEE in our series, while younger patients and those with previous mild hepatic disease were at higher risk (Table 5). After adjustment for confounders, only diabetes mellitus with end-organ damage maintained a significant aHR.

## 4. Discussion

In this study, we confirmed that LEE were associated with mortality and ICU admission among COVID-19 patients in a large cohort of European patients. In addition, we found that even low-grade LEE were associated with these outcomes, and that the association became stronger for higher levels of LEE, with grade 4 LEE predicting the risk of death. These results are of great importance, since AST and ALT evaluation are low-cost tests, available in almost all hospitals, and can be performed in resource-limited settings. Moreover, to date, this is one of the largest series described in Europe, and the implications of this study’s results could be useful in everyday clinical practice, where patients with LEE could be considered for hospital admission and intermediate to high intensity monitoring, even in the absence of baseline comorbidities or other factors predictive of poor outcomes. Although the association between LEE and poorer outcome is now consolidated and supported by evidence on large cohorts of patients [17,18,20,25,26], the reasons for this are still to be clarified. In fact, LEE have been proposed to be innocent bystanders [32] of a systemic damage, while deaths linked to liver insufficiency have been rarely reported in the course of COVID-19 [33,34]. Although we found several factors associated with LEE, they showed an inverse association with factors commonly related to poor prognosis, such as older age [15], diabetes [35] or chronic kidney disease [36]. These findings are in accordance with previous studies, which found LEE to be less frequent in diabetic patients [25,26] and more frequent in younger patients [25] with COVID-19. A possible explanation for this could be that, at least in a proportion of patients, LEE are the expression of a more robust immune and inflammatory response to infection mounted by younger patients and resulting in a complex host–immune interaction, with possible immune-mediated liver injury. This hypothesis could be supported by the fact that in our study population, baseline CRP and ferritin levels correlated with LEE by univariate analysis and markers of inflammation have been linked with LEE also in previous works [25]. On the other hand, the immune response could be milder in diabetic or nephropathic patients, in whom AST and ALT levels have been reported to be significantly lower than in other patients, even in contexts different from SARS-CoV-2 infection [37]. Even if the immune-mediated liver damage is a fascinating hypothesis to explain LEE, it cannot be the only explanation of the phenomenon. In fact, we also found that patients exposed to different drugs were at higher risk of LEE. In particular, although all the studied drugs showed an increased hazard ratio of LEE by univariate analysis, only darunavir/ritonavir, tocilizumab and steroid use maintained a significance also after adjustment for possible confounding factors. All these drugs have known potential liver toxicity [30,38,39,40]; it is possible that they were used more frequently in patients with more severe disease and that LEE could be the result of the expression of both drug-induced injury and liver injury secondary to a more severe systemic condition in these patients. Indeed, in our series, patients with higher respiratory rate upon first clinical presentation were at higher risk of LEE, suggesting that, at least in some patients, LEE might be the expression of a more severe systemic disease implying hepatic hypoxia or congestion. Finally, LEE occurred more frequently in patients with pre-existing liver disease or with other markers of liver injury, such as low albumin or higher GGT, supporting the hypothesis of a liver origin of transaminase release instead of a muscular one.

The limitations of the present study are the observational design, that, despite the multivariable analysis being corrected for multiple confounders, cannot exclude residual confounding. For the same reason, we can only observe the association between LEE and the clinical and laboratory characteristics of the study population but cannot add insights on the causality of LEE. Moreover, the choice of studying LEE grade ≥2 could underestimate the number of clinical events, since we have not considered study participants with transaminase levels next to normal, but formally falling into the category of grade 1 LEE. Also, the body mass index of the study participants was not available, so we could not investigate the role of being overweight or obese on LEE occurrence or disease severity. Importantly, the lack of data about blood or intra-hepatic levels of SARS-CoV-2 RNA keeps us from correlating our results with the viral burden and thus distinguishing between direct virus damage or secondary injury linked to an excessive immune response. Finally, the enrolled patients represent an almost exclusively inpatient population, and the study period includes a moment when in our center, only the sickest patients were tested for SARS-CoV-2. Therefore, this information may not be applicable to outpatients or to patients with mild disease. In conclusion, we found that in a large cohort of European patients, a simple, inexpensive, and widely available blood test such as transaminase evaluation can offer important information about the prognosis of patients with COVID-19. Grade ≥2 LEE was associated with the risk of a more severe clinical course in terms of ICU admission and/or death, and grade 4 LEE predicted mortality. The origin of LEE is probably multifactorial and may be linked to viral hepatitis, drug toxicity, and immune system overreaction. Transaminase release could also actually be an innocent bystander of a more complex systemic damage, but is easy to detect and has a prognostic value that has been confirmed in multiple studies in different populations [8,16,17,18,20,21,25,26]. However, the fact that not only patients with other known negative prognostic factors develop LEE suggests that LEE evaluation itself could add information to clinical variables that are commonly evaluated in the course of COVID-19.

## Figures and Tables

**Table 1:** Definition of liver enzyme elevation (LEE). Patients were classified based on changes relative to the upper limit of normal (ULN) of the reference laboratory, i.e., 40 U/L for both AST and ALT.

Absence of LEE	Both AST and ALT Values <1.25 ULN
LEE grade 1	AST and/or ALT between 1.25–2.4× ULN
LEE grade 2	AST and/or ALT between 2.5–5× ULN
LEE grade 3	AST and/or ALT between 5.1–10× ULN
LEE grade 4	AST and/or ALT >10× ULN

AST: aspartate aminotransferase; ALT: alanine aminotransferase; ULN: upper limit of normal.

**Table 2:** Baseline characteristics of patients included in the study according to liver enzyme elevation (LEE) grade ≥2 development in the course of COVID-19.

	Whole Population (*n* = 799)		Patients without LEE (*n* = 574)		Patients with LEE (*n* = 225)		
Basline Characteristics	*n*	Mean (SD) or Frequency (%)	*n*	Mean (SD) or Frequency (%)	*n*	Mean (SD) or Frequency (%)	*p*-value
Age (years), mean (SD)	799	69.93 (16.0)	574	71.44 (16.5)	225	66.07 (14.0)	<0.0001
Male sex, N (%)	799	480 (60.0)	574	315 (58.4)	225	165 (73.3)	<0.0001
Weight (kg), mean (SD)	36	76.58 (19.7)	23	75.43 (22.9)	13	78.62 (13.1)	0.276
Charlson Index, mean (SD)	602	4.18 (3.1)	415	4.54 (3.2)	187	3.37 (2.7)	<0.0001
Hypertension, N (%)	679	332 (48.9)	472	238 (50.4)	207	94 (45.4)	0.229
Diabetes, N (%)	678	104 (15.3)	471	82 (17.4)	207	22 (10.6)	0.024
Ischemic heart disease, N (%)	673	64 (9.5)	576	46 (9.8)	206	18 (8.7)	0.650
Peripheral vasculopathy, N (%)	666	131 (19.6)	461	106 (23.0)	205	25 (12.2)	0.001
Cerebrovascular disease, N (%)	667	92 (13.8)	461	75 (16.3)	206	17 (8.3)	0.006
COPD, N (%)	669	77 (11.5)	466	59 (12.8)	203	18 (8.9)	0.158
Cancer, N (%)	669	74 (11.1)	463	59 (12.7)	206	15 (7.8)	0.038
CKD, N (%)	662	72 (10.9)	461	65 (14.1)	201	7 (3.5)	<0.0001
Dementia, N (%)	667	69 (10.3)	460	59 (12.8)	207	10 (4.8)	0.002
Mild liver disease, N (%)	666	19 (2.9)	460	9 (2.0)	206	10 (4.9)	0.038
Moderate/severe liver disease, N (%)	671	11 (1.6)	465	7 (1.5)	206	4 (1.9)	0.744
Parameters at First Clinical Presentation							
Respiratory rate, mean (SD)	317	21.33 (8.4)	200	20.93 (9.2)	117	22.01 (6.8)	0.091
PaO_2_/FiO_2,_ mean (SD)	470	228.7 (707.5)	297	213.7 (688.3)	173	254.6 (740.6)	0.028
WBC (×10^9^/L), mean (SD)	766	7.61 (4.4)	548	7.43 (3.9)	218	8.08 (5.6)	0.282
Lymphocytes (×10^9^/L), mean (SD)	680	1.07 (1.8)	481	1.02 (09)	199	1.19 (2.9)	0.787
PTL (×10^9^/L), mean (SD)	766	208.62 (96.9)	548	211.63 (99.3)	218	201.06 (90.4)	0.272
FIB4, mean (SD)	637	3.53 (5.1)	446	3.18 (3.7)	191	4.34 (7.4)	0.001
ALT (U/L), mean (SD)	738	43.32 (69.1)	523	30.09 (17.0)	215	75.51 (119.5)	<0.0001
AST (U/L), mean (SD)	638	48.79 (64.0)	447	35.06 (18.6)	191	80.90 (106.9)	<0.0001
GGT (U/L), mean (SD)	724	77.12 (119.5)	512	55.79 (70.6)	212	128.64 (181.9)	<0.0001
Bilirubin (mg/dL), mean (SD)	720	0.60 (0.5)	502	0.58 (0.4)	218	0.65 (0.6)	0.006
INR, mean (SD)	715	1.27 (0.4)	502	1.28 (0.4)	213	1.25 (0.2)	0.231
Ferritin, mean (SD)	592	928.66 (946.9)	404	738.17 (749.4)	188	1338.02 (1173.3)	<0.0001
IL-6, mean (SD)	580	118.24 (456.5)	392	126.83 (492.2)	188	100.31 (371.7)	0.003
Albumin, mean (SD)	268	27.90 (6.4)	170	28.47 (6.5)	98	26.92 (6.3)	0.054
CRP, mean (SD)	760	91.90 (84.1)	542	83.91 (78.2)	218	111.75 (94.4)	<0.0001
Hospital stay (days), mean (SD)	691	16.64 (16.6)	490	15.06 (16.6)	201	20.48 (15.8)	<0.0001
Drug Use							
ARBs *	653	95 (14.5)	450	66 (14-7)	203	29 (14.3)	0.898
ACEIs *	654	100 (15.3)	451	69 (15.3)	203	31 (15.3)	0.993
NSAIDs *	644	20 (3.1)	446	15 (3.4)	198	5 (2.5)	0.572
Steroids **	799	342 (42.8)	574	208 (36.2)	225	134 (59.6)	<0.0001
Remdesivir **	799	6 (0.7)	574	3 (0.5)	225	3 (1.3)	0.358
Antibiotics **	799	419 (52.4)	574	257 (44.8)	225	162 (72.0)	<0.0001
HCQ **	799	469 (58.7)	574	292 (50.9)	225	177 (78.7)	<0.0001
LPV/r **	799	4 (0.5)	574	3 (0.5)	225	1 (0.4)	1.0
DRV/r **	799	162 (20.3)	574	90 (15.7)	225	72 (32.0)	<0.0001
Tocilizumab **	799	164 (20.5)	574	61 (10.6)	225	103 (45.8)	<0.0001

Abbreviations: ACEIs: ACE inhibitors; ALT: alanine aminotransferase; AST: aspartate aminotransferase; ARBs: angiotensin receptor antagonists; CKD: chronic kidney disease; COPD: chronic obstructive pulmonary disease; CRP: C reactive protein; DRV/r: darunavir/ritonavir; GGT: gamma-glutamyltransferase; HCQ: hydroxychloroquine; IL-6: interleukin-6; LEE: liver enzyme elevation; LPV/r: lopinavir/ritonavir; n: number of patients with available data; NSAIDs: Non-steroidal anti-inflammatory drugs; PLT: platelets; SD: standard deviation; WBC: white blood cells. Significant *p* values (<0.05) are indicated in bold. * Chronic treatment, ** Treatment in course of hospital stay.

**Table 3:** Crude (HR) and adjusted hazard ratio (aHR) for death and intensive care unit (ICU) admission of clinical and laboratory factors at first clinical presentation in the study population.

Parameter	HR	95% CI		*p*	aHR	95% CI		*p*
Age	1.04	1.03	1.04	<0.0001	1.03	1.02	1.04	<0.0001
Male sex	1.53	1.22	1.92	0.0002	1.32	1.04	1.68	0.021
Weight	1.00	1.00	1.01	0.100				
Charlson Comorbidity Index	1.12	1.09	1.15	<0.0001	1.07	1.04	1.12	0.000
Respiratory rate	1.01	1.00	1.01	0.002				
PaO_2_/FiO_2_	1.00	1.00	1.00	0.520				
Hypertension	1.45	1.25	1.67	<0.0001				
Diabetes	1.26	1.09	1.45	0.002				
COPD	1.23	1.02	1.48	0.032				
Mild liver disease	1.94	1.17	3.21	0.010				
Moderate to severe liver disease	2.19	1.27	3.78	0.005				
CKD	1.56	1.26	1.92	<0.0001				
ARBs *	1.12	0.91	1.37	0.289				
ACEIs *	1.10	0.75	1.61	0.639				
NSAIDs *	1.26	1.04	1.54	0.021				
Length of hospital stay	1.00	1.00	1.00	0.038				
Laboratory								
LEE (grade ≥ 2)	1.46	1.17	1.82	0.001	1.46	1.14	1.88	0.003
WBC	1.07	1.06	1.09	<0.0001	1.05	1.03	1.07	<0.0001
Lymphocytes	0.95	0.86	1.06	0.358				
PTL	1.00	1.00	1.00	0.101				
GGT	1.00	1.00	1.00	0.042				
Total bilirubin	1.32	1.15	1.52	0.0001				
Prothrombin time	1.46	1.22	1.75	<0.0001	1.37	1.09	1.72	0.006
Albumin	0.92	0.90	0.94	<0.0001				
Ferritin	1.00	1.00	1.00	<0.0001	1.00	1.00	1.00	0.029
IL-6	1.00	1.00	1.00	<0.0001	1.00	1.00	1.00	0.001
CRP	1.01	1.00	1.01	<0.0001	1.00	1.00	1.01	<0.0001
Drug use								
Steroids	0.78	0.63	0.97	0.026	0.73	0.58	0.91	0.005
Remdesivir	2.75	1.14	6.65	0.025				
Antibiotics	1.22	0.98	1.51	0.072				
HCQ	0.90	0.72	1.11	0.320				
LPV/r	2.11	0.68	6.57	0.198				
DRV/r	1.39	1.09	1.78	0.008	1.32	1.01	1.71	0.039
Tocilizumab	0.89	0.68	1.16	0.387				

The multivariable model has been adjusted for age, sex, Charlson Comorbidity Index, respiratory rate, hypertension, LEE grade ≥ 2, length of hospital stay, baseline weight, chronic ACEIs use, PaO_2_/FiO_2_, WBC, GGT, total bilirubin, albumin, prothrombin time, ferritin, IL-6, CRP, DRV/r, Remdesivir, antibiotics and steroid use. Abbreviations: 95% CI: 95% confidence interval; ACEIs: ACE inhibitors; aHR: adjusted hazard ratio; ALT: alanine aminotransferase; AST: aspartate aminotransferase; ARBs: angiotensin receptor antagonists; CKD: chronic kidney disease; COPD: chronic obstructive pulmonary disease; CRP: C reactive protein; DRV/r: darunavir/ritonavir; GGT: gamma-glutamyltransferase; HR: hazard ratio; HCQ: hydroxychloroquine; IL-6: interleukin-6; LEE: liver enzyme elevation; LPV/r: lopinavir/ritonavir; NSAIDs: non-steroidal anti-inflammatory drugs; PLT: platelets; WBC: white blood cells. * Chronic therapy.

**Table 4:** Crude (HR) and adjusted hazard ratio (aHR) for LEE grade ≥2.

Parameter	HR	95% CI		*p*	aHR	95% CI		*p*
Age	0.99	0.98	1.00	0.0157				
Male sex	2.32	1.71	3.15	<0.0001	1.73	1.26	2.38	0.0007
Weight	1.00	1.00	1.01	0.330				
Charlson Comorbidity Index	0.90	0.86	0.95	<0.0001	0.93	0.88	0.98	0.0096
Respiratory rate	1.01	1.00	1.02	0.002	1.01	1.00	1.02	0.0122
PaO_2_/FiO_2_	1.00	1.00	1.00	0.162				
Hypertension	0.90	0.69	1.17	0.414				
ARBs *	1.22	0.84	1.79	0.300				
ACEIs *	1.33	0.93	1.92	0.123				
NSAIDs *	0.92	0.41	2.07	0.837				
Length of hospital stay	1.01	1.00	1.02	0.001				
Laboratory								
WBC	1.04	1.02	1.07	0.001				
PTL	1.00	1.00	1.00	0.185				
GGT	1.00	1.00	1.00	<0.0001	1.00	1.00	1.00	<0.0001
Total bilirubin	1.31	1.04	1.63	0.019				
Prothrombin time	0.78	0.51	1.20	0.257				
Albumin	0.93	0.90	0.95	<0.0001	0.95	0.93	0.98	0.0002
Ferritin	1.00	1.00	1.00	<0.0001	1.00	1.00	1.00	<0.0001
IL-6	1.00	1.00	1.00	0.269				
CRP	1.01	1.00	1.01	<0.0001				
Drug use								
Steroids	2.02	1.54	2.65	<0.0001	1.77	1.33	2.36	0.0001
Remdesivir	1.57	0.50	4.92	0.436				
Antibiotics	2.51	1.86	3.39	<0.0001				
HCQ	2.74	1.96	3.83	<0.0001				
LPV/r	1.09	0.15	7.74	0.933				
DRV/r	1.96	1.48	2.60	<0.0001	1.51	1.12	2.03	0.0063
Tocilizumab	3.79	2.90	4.94	<0.0001	2.06	1.54	2.76	<0.0001

The multivariable model has been adjusted for age, sex, Charlson Comorbidity Index, Respiratory rate, length of hospital stay, WBC, GGT, total bilirubin, albumin, ferritin, CRP, DRV/r, HCQ, tocilizumab, antibiotics and steroid use. Abbreviations: 95% CI: 95% confidence interval; ACEIs: ACE inhibitors; aHR: adjusted hazard ratio; ALT: alanine aminotransferase; AST: aspartate aminotransferase; ARBs: angiotensin receptor antagonists; CRP: C reactive protein; DRV/r: darunavir/ritonavir; GGT: gamma-glutamyltransferase; HR: hazard ratio; HCQ: hydroxychloroquine; IL-6: interleukin-6; LEE: liver enzyme elevation; LPV/r: lopinavir/ritonavir; NSAIDs: non-steroidal anti-inflammatory drugs; PLT: platelets; WBC: white blood cells. * Chronic therapy.

**Table 5:** Crude (HR) and adjusted hazard ratio (aHR) for LEE grade ≥2 according to baseline comorbidities.

Parameter	HR	95% CI		*p*	aHR	95% CI		*p*
Age	0.99	0.98	1.00	0.0157				
Myocardial infarction	1.01	0.62	1.66	0.9651				
CHF	0.94	0.55	1.58	0.8027				
Peripheral vascular disease	0.57	0.38	0.86	0.0068				
CVA or TIA	0.78	0.49	1.24	0.2918				
Dementia	0.57	0.30	1.08	0.0833				
COPD	1.06	0.68	1.65	0.7847				
Connective tissue disease	0.77	0.19	3.10	0.7132				
Peptic ulcer disease	0.76	0.28	2.04	0.5865				
Mild liver disease	2.75	1.46	5.19	0.0018				
Moderate to severe liver disease	1.08	0.35	3.37	0.8957				
Diabetes (uncomplicated)	0.79	0.51	1.22	0.2870				
Diabetes (end-organ damage)	0.39	0.15	1.05	0.0613	0.32	0.12	0.88	0.027
Hemiplegia	0.87	0.28	2.72	0.8117				
CKD	0.50	0.26	0.94	0.0310				
Solid tumor	0.75	0.45	1.24	0.2582				
Leukemia	1.54	0.57	4.14	0.3924				
Lymphoma	0.98	0.24	3.93	0.9737				
AIDS	0.00	0.00	3.82	0.9709				

The multivariable model has been adjusted for age, sex, peripheral vascular disease, dementia, diabetes with end-organ damage, CKD, respiratory rate, length of hospital stay, WBC, GGT, total bilirubin, albumin, ferritin, CRP, DRV/r, HCQ, tocilizumab, antibiotics and steroid use. Table legend: 95% CI: 95% confidence interval; aHR: adjusted hazard ratio; CHF: chronic heart failure; COPD: chronic obstructive pulmonary disease; CVA: cerebrovascular accident; TIA: transient ischemic attack; CKD: chronic kidney disease; AIDS: acquired immunodeficiency syndrome.

## Supplementary Materials

The following are available online at https://www.mdpi.com/2076-2607/8/12/2010/s1, Table S1: Crude (HR) and adjusted hazard ratio (aHR) for death in the study population according to grade 4 liver enzyme elevation (LEE), Table S2: Median levels of aspartate aminotransferase (AST) and alanine aminotransferase (ALT) at first clinical presentation in people who developed or did not develop liver enzyme elevation (LEE). Table S3: Crude (HR) and adjusted hazard ratio (aHR) for death and ICU admission of clinical and laboratory factors at first clinical presentation in the study population. Patients for whom data were imputed were excluded in this analysis.
