# Supplementary material for: Higher Mortality and Intensive Care Unit Admissions in COVID-19 Patients with Liver Enzyme Elevations
Source: Microorganisms. 2020 Dec 16;8(12):2010. doi: 10.3390/microorganisms8122010 (PMC7766471; doi:10.3390/microorganisms8122010)
Supplement: Supplementary file 1 [file microorganisms-08-02010-s001.pdf]

Supplementary Table 1. Crude (HR) and adjusted hazard ratio (aHR) for death in the study population according to grade 4 liver enzyme elevation (LEE).

Parameter	HR	95% CI		*p*	aHR	95% CI		*p*
**Age**	**1.06**	1.05	1.07	<.0001	1.05	1.04	1.06	<.0001
Male sex	1.34	1.05	1.71	0.018	1.44	1.11	1.87	0.006
Weight	1.00	1.00	1.01	0.242				
Charlson Comorbidity Index	1.18	1.15	1.21	<.0001	1.10	1.06	1.14	<.0001
Respiratory rate	1.01	1.00	1.01	0.146				
PaO2/FiO2	1.00	1.00	1.00	0.669				
Hypertension	1.60	1.39	1.84	<.0001				
Diabetes	1.35	1.18	1.54	<.0001				
COPD	1.31	1.09	1.57	0.003				
Mild liver disease	2.21	1.31	3.73	0.003				
Moderate to severe liver disease	2.45	1.38	4.36	0.002				
CKD	1.80	1.48	2.19	<.0001				
ARBs **	1.15	0.933	1.44	0.194				
ACEIs **	1.33	1.09	1.63	0.005				
NSAIDs **	1.25	0.88	1.76	0.210				
Length of hospital stay	1.00	1.00	1.00	0.850				
**Laboratory**								
LEE (grade 4*)	1.529	0.858	2.725	0.1501	2.64	1.43	4.88	0.002
WBC	1.051	1.033	1.069	<.0001				
Lymphocytes	0.718	0.596	0.865	0.0005				
PTL	0.998	0.997	1.000	0.0117				
GGT	1.000	0.999	1.001	0.7002				
Total bilirubin	1.413	1.219	1.637	<.0001				
Prothrombin time	1.465	1.219	1.759	<.0001	1.36	1.07	1.73	0.013
Albumin	0.931	0.913	0.951	<.0001				
Ferritin	1.000	1.000	1.000	<.0001	1.00	1.00	1.00	0.012
IL-6	1.000	1.000	1.001	<.0001	1.00	1.00	1.00	0.011
CRP	1.005	1.003	1.006	<.0001	1.01	1.00	1.01	<.0001
**Drug use**								
Steroids	0.584	0.457	0.745	<.0001	0.61	0.47	0.79	<.0001
Remdesivir	0.777	0.193	3.12	0.7217				
Antibiotics	0.848	0.673	1.068	0.1615				
HCQ	0.577	0.458	0.727	<.0001				
LPV/r	2.801	0.897	8.741	0.0761	3.73	1.18	11.83	0.025
DRV/r	0.862	0.642	1.156	0.3217				
Tocilizumab	0.484	0.342	0.683	<.0001	0.58	0.39	0.85	0.006

The multivariable model has been adjusted for age, sex, Charlson Comorbidity Index, hypertension, LEE grade 4, chronic ACEI use, WBC, lymphocytes, PTL, total bilirubin, albumin, prothrombin time, ferritin, IL-6, CRP, steroids, HCQ, LPV/r and tocilizumab use. Abbreviations: 95% CI: 95% confidence interval; ACEIs: ACE inhibitors; aHR: adjusted hazard ratio; ALT: alanine aminotransferase; AST: aspartate aminotransferase; ARBs: angiotensin receptor antagonists; CKD: chronic kidney disease; COPD: chronic obstructive pulmonary disease; CRP: C reactive protein; DRV/r: darunavir/ritonavir; GGT: gamma-glutamyltransferase; HR: hazard ratio; HCQ: hydroxychloroquine; IL-6: interleukin-6; LEE: liver enzyme elevation; LPV/r: lopinavir/ritonavir; NSAIDs: non-steroidal anti-inflammatory drugs; PLT: platelets; WBC: white blood cells. *Number of events in the study population = 22. ** Chronic treatment.

Supplementary Table 2. Median levels of aspartate aminotransferase (AST) and alanine aminotransferase (ALT) at first clinical presentation in people who developed or did not develop liver enzyme elevation (LEE).

	LEE grade ≥2	*p* value

	No	Yes	
AST* U/L; median (IQR)	26 (20–36)	50 (33.5–78)	0.0001
ALT* U/L; median (IQR)	23 (17–32)	41 (29–68)	0.0001
	LEE grade ≥3		
	No	Yes	
AST* U/L; median (IQR)	32 (22–48)	54 (35¬102)	0.0001
ALT* U/L; median (IQR)	27 (19.5–39.5)	48 (31–82)	0.0001
	LEE grade 4		
	No	Yes	
AST* U/L; median (IQR)	29 (21–44)	48 (30–84)	0.0001
ALT* U/L; median (IQR)	34 (24–52)	53 (32–119)	0.0001

* Liver enzymes at fist clinical presentation. IQR: interquartile range

Supplementary Table 3. Crude (HR) and adjusted hazard ratio (aHR) for death and ICU admission of clinical and laboratory factors at first clinical presentation in the study population. Patients for whom data were imputed were excluded in this analysis.

Parameter	HR	95% CI		*p*	aHR	95% CI		*p*
**Age**	**1.04**	1.03	1.04	<.0001				
Male sex	1.53	1.22	1.92	0.001				
Respiratory rate	1.02	1.00	1.03	0.018				
Hypertension	1.51	1.20	1.90	0.001				
Diabetes	1.43	1.07	1.90	0.016				
COPD	1.23	0.88	1.73	0.229				
Mild liver disease	1.90	1.07	3.39	0.029				
Moderate to severe liver disease	1.90	1.07	3.39	0.029				
CKD	1.83	1.33	2.51	0.001				
Length of hospital stay	1.01	1.00	1.01	0.077				
**Laboratory**								
LEE (grade >2)	1.458	1.167	1.822	0.0009				
WBC	1.074	1.058	1.089	<.0001	1.11	1.03	1.20	0.0100
Lymphocytes	0.971	0.877	1.075	0.5664				
PTL	0.999	0.998	1.000	0.1002				
GGT	1.001	1.000	1.002	0.0341				
Total bilirubin	1.445	1.245	1.676	<.0001				
Prothrombin time	1.480	1.216	1.802	<.0001				
Albumin	0.937	0.914	0.960	<.0001				
Ferritin	1.000	1.000	1.000	<.0001	1.00	1.00	1.00	0.0020
IL-6	1.000	1.000	1.001	<.0001	1.00	1.00	1.01	0.0004
CRP	1.005	1.004	1.006	<.0001				
**Drug use**								

Steroids	0.782	0.63	0.971	0.0258				
Remdesivir	2.750	1.137	6.652	0.0248				
Antibiotics	1.216	0.983	1.506	0.0719				
HCQ	0.897	0.725	1.111	0.3200				
LPV/r	2.110	0.677	6.573	0.1977				
DRV/r	1.394	1.091	1.781	0.0079	4.42	2.07	9.47	0.0001
Tocilizumab	0.889	0.681	1.161	0.3873				

The multivariable model has been adjusted for age, sex, Charlson Comorbidity Index, respiratory rate, hypertension, LEE of grade ≥2, length of hospital stay, WBC, GGT, total bilirubin, albumin, prothrombin time, ferritin, IL-6, CRP, DRV/r, remdesivir, tocilizumab, antibiotics and steroid use. In the multivariable model, 54/799 patients were retained. Table legend: 95% CI: 95% confidence interval; aHR: adjusted hazard ratio; ALT: alanine aminotransferase; AST: aspartate aminotransferase; CKD: chronic kidney disease; COPD: chronic obstructive pulmonary disease; CRP: C reactive protein; DRV/r: darunavir/ritonavir; GGT: gamma-glutamyltransferase; HR: hazard ratio; HCQ: hydroxychloroquine; IL-6: interleukin-6; LEE: liver enzyme elevation; LPV/r: lopinavir/ritonavir; PLT: platelets; WBC: white blood cells.
